# Annotation of primate miRNAs by high throughput sequencing of small RNA libraries

**DOI:** 10.1186/1471-2164-13-116

**Published:** 2012-03-27

**Authors:** Michael Dannemann, Birgit Nickel, Esther Lizano, Hernán A Burbano, Janet Kelso

**Affiliations:** 1 Max Planck Institute for Evolutionary Anthropology, Department of Evolutionary Genetics, Deutscher Platz 6, Leipzig 04103, Germany; 2 Center for Genomic Regulation, Department of Genetic Causes of Disease, C/Dr. Aiguader 88, 08003 Barcelona, Spain; 3 Current address: Max Planck Institute for Developmental Biology, Department of Molecular Biology, Spemannstrasse 37-39, Tübingen 72076, Germany

## Abstract

**Background:**

In addition to genome sequencing, accurate functional annotation of genomes is required in order to carry out comparative and evolutionary analyses between species. Among primates, the human genome is the most extensively annotated. Human miRNA gene annotation is based on multiple lines of evidence including evidence for expression as well as prediction of the characteristic hairpin structure. In contrast, most miRNA genes in non-human primates are annotated based on homology without any expression evidence. We have sequenced small-RNA libraries from chimpanzee, gorilla, orangutan and rhesus macaque from multiple individuals and tissues. Using patterns of miRNA expression in conjunction with a model of miRNA biogenesis we used these high-throughput sequencing data to identify novel miRNAs in non-human primates.

**Results:**

We predicted 47 new miRNAs in chimpanzee, 240 in gorilla, 55 in orangutan and 47 in rhesus macaque. The algorithm we used was able to predict 64% of the previously known miRNAs in chimpanzee, 94% in gorilla, 61% in orangutan and 71% in rhesus macaque. We therefore added evidence for expression in between one and five tissues to miRNAs that were previously annotated based only on homology to human miRNAs. We increased from 60 to 175 the number miRNAs that are located in orthologous regions in humans and the four non-human primate species studied here.

**Conclusions:**

In this study we provide expression evidence for homology-based annotated miRNAs and predict *de novo *miRNAs in four non-human primate species. We increased the number of annotated miRNA genes and provided evidence for their expression in four non-human primates. Similar approaches using different individuals and tissues would improve annotation in non-human primates and allow for further comparative studies in the future.

## Background

From a comparative genomics standpoint the great apes are among the most studied groups of organisms [1]. Since the completion of human genome sequencing in 2001 [2,3] the genomes of all species belonging to this family have been or are being sequenced [4,5]. Although only the human reference genome is considered of finished quality [2,3], it is possible to compare and also use these genomes sequences as references for the alignment of reads generated in sequencing and gene expression studies. In addition to determine the DNA sequence of a genome, it is of particular importance to attach biological information to it e.g. determine the location and structure of protein-coding genes. Gene annotation is carried out both computationally and experimentally by sequencing cDNA e.g. traditionally using expressed sequence tags (ESTs) [6,7] and more recently RNA-seq [8]. Human EST resources are also more abundant than their non-human counterparts and therefore human gene annotation is also the most accurate among great apes [9]. While the majority of efforts have focused on the annotation of protein-coding genes, the discovery of large-scale transcription outside of protein-coding genes [10,11] has led to the identification of a great diversity of non-protein-coding RNA genes [12]. Among these are the microRNAs (miRNAs) which are short (~22 bp) RNA molecules [13] that post-transcriptionally down-regulate protein-coding gene expression [14,15]. The official repository of miRNAs miRBase (v.17) [16,17] contains 1,424 human miRNA, whereas fewer miRNAs are annotated in other primate genomes (chimpanzee: 600; bonobo: 88; gorilla: 85; orangutan: 581; rhesus macaque: 479), a fact that is explained by the larger number of human studies.

MiRNAs have been annotated in humans using a mixture of bioinformatics prediction and cDNA sequencing [18]. The identification of miRNAs in non-human primates has made use of a number of comparative methodologies such as sequence homology between closely related organisms [19-22], the genomic search for RNA secondary structure patterns characteristic of miRNAs [23] and by direct sequencing of small RNA libraries [24,25]. However, direct characterization of small RNA libraries by high throughput sequencing has been performed for a limited number of tissues in only chimpanzees and rhesus macaques[24,25]. As a result the majority of non-human primate miRNAs in miRBase have no evidence for their expression and their existence is only supported by computational prediction. In the present study we sequenced small RNA libraries from multiple chimpanzee, gorilla, orangutan and rhesus macaque individuals and tissues using the Illumina high throughput sequencing platform. We applied an algorithm (miRDeep) that uses sequencing reads in conjunction with a model of miRNA biogenesis to predict miRNAs with high accuracy[26,27].

## Results

### MiRNA prediction

**Figure 1:**
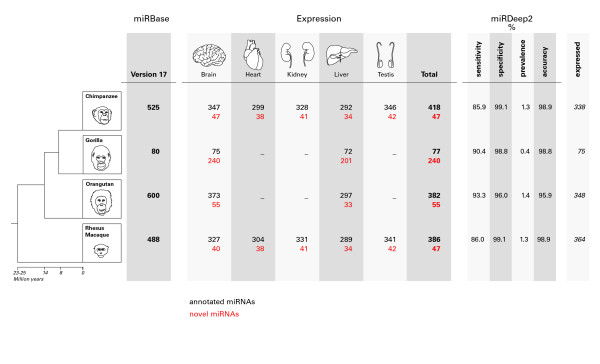
**Expression of annotated and novel miRNAs for the four primate species**. Column 1 illustrates the number of annotated miRBase (version 17) miRNAs. Columns 2-6 contain the number of expressed annotated (black) and novel (red) miRNAs for each separate tissue and column 7 for the union of all tissues. Columns 8-11 show miRDeep2 statistics and column 12 the number of miRNAs miRDeep2 defined as expressed and calculated its summary statistics on.

We used the program miRDeep2 [27] to predict miRNAs from sequenced small RNAs. miRDeep2 takes as input the position and frequency of reads aligned to the genome ("signature") with respect to a putative RNA hairpin and scores the miRNA candidate employing a probabilistic model based on miRNA biogenesis [26]. The score produced by miRDeep takes into account the energetic stability of the putative hairpin and the compatibility of the observed read distribution with miRNA cleavage [26]. The more positive the score the more reliable the prediction. Additionally, miRDeep2 calculates false-positive rates by running the algorithm on a set of "signatures" and secondary structures that are paired by random permutation. Using predictions with a positive score and a significant folding p-value we identified from our sequences 47 (22 with expression evidence for star sequence) new miRNAs in chimpanzee, 240 (166 with expression evidence for star sequence) in gorilla, 55 (13 with expression evidence for star sequence) in orangutan and 47 (24 with expression evidence for star sequence) in rhesus macaque. miRDeep2 was able to predict 338 (64% of all annotated) known miRNAs (312 with a positive score) in chimpanzee, 75 (94% of all annotated, 73 with a positive score) in gorilla, 364 (61% of all annotated, 325 with a positive score) in orangutan and 348 (71% of all annotated, 312 with a positive score) in rhesus macaque (Figure 1). miRDeep2 performance statistics were similar to the ones reported in other species [27] (Figure 1).

MiRNAs show high expression conservation between species, and tissue-specific expression patterns [28,29]. In testis we found a lower fraction of the total reads align to miRNAs (Table 1) as a result of the expression of an additional class of small-RNAs in this tissue - piRNAs [29]. We were able to identify 11 tissue-specific miRNAs in chimpanzee (7 in brain, 1 in heart, 2 in kidney, 1 in testis), 110 in gorilla (100 in brain, 10 in liver), 28 in orangutan (25 in brain, 3 in liver) and 21 in rhesus macaque (11 in brain, 10 in testis).

**Table 1:** Samples' read alignment information.

Individual	Tissue	Genome	miRBase miRNAs	Predictions	Unknown	Total reads
Chimp 1	Brain	42.3	78.8	2.9	18.3	12211879
Chimp 2	Brain	54.3	90	2.2	7.7	11658357
Chimp 3	Brain	54.8	73.5	2.4	24.2	8627942
Chimp 4	Brain	52.5	88.1	2.3	9.6	10381037
Chimp 5	Brain	18.7	79.4	2.2	18.4	13977547
Chimp 1	Liver	57.4	92.3	1.1	6.6	8262666
Chimp 2	Liver	63.9	89.3	0.9	9.8	8088806
Chimp 3	Liver	51.7	88.1	0.9	11	11017642
Chimp 4	Liver	52.3	93.8	1.1	5.1	10449677
Chimp 5	Liver	29.9	57.5	0.5	41.9	16283995
Chimp 2	Testis	49.1	4.2	1.6	94.2	11361816
Chimp 3	Testis	63	5.8	2.1	92	8899032
Chimp 4	Testis	40.7	8.6	3.4	88	11965804
Chimp 5	Testis	43.2	5.8	2.1	92.1	11875495
Chimp 6	Testis	51.3	6.8	4	89.2	11166737
Chimp 1	Kidney	60.3	91	2.8	6.2	9702033
Chimp 2	Kidney	44.5	83.3	2.8	13.9	7774225
Chimp 3	Kidney	61.6	86.4	3.4	10.2	10250184
Chimp 5	Kidney	57.9	83.4	3.6	12.9	10264521
Chimp 1	Heart	63.2	94.7	2.2	3.1	7818504
Chimp 2	Heart	63.6	96.4	1.5	2	8644295
Chimp 3	Heart	65.4	95.3	1.1	3.6	9426585
Chimp 4	Heart	61.3	88	1.6	10.5	9449302
Chimp 5	Heart	60.8	88	1.3	10.7	9124991
Rhesus 1	Brain	36.1	72.3	4.8	23	12946219
Rhesus 2	Brain	38	81.8	4.5	13.7	12258382
Rhesus 3	Brain	47.5	82.3	5.6	12.1	11623674
Rhesus 4	Brain	44.9	90.6	3.6	5.8	11490940
Rhesus 5	Brain	48.9	88.4	3.8	7.8	10898842
Rhesus 1	Liver	51.4	93.4	1.3	5.4	8615049
Rhesus 2	Liver	58.2	95.1	1.1	3.8	8617533
Rhesus 3	Liver	54.7	95	2	3	9668109
Rhesus 4	Liver	45.6	94.6	1.8	3.7	10620490
Rhesus 5	Liver	34.5	90.6	1.9	7.5	10750399
Rhesus 1	Testis	44.4	36.3	1.1	62.5	12068068
Rhesus 2	Testis	25.7	40.2	2.7	57	14533174
Rhesus 3	Testis	47	29.9	1.1	69.1	11467601
Rhesus 4	Testis	50.5	15.2	0.4	84.3	10760301
Rhesus 1	Kidney	39.5	59.4	1.3	39.2	10730625
Rhesus 2	Kidney	52.4	87.9	2.4	9.6	12158274
Rhesus 3	Kidney	58.5	86.5	2.8	10.7	10683932
Rhesus 4	Kidney	55.8	81.5	2.3	16.2	10704780
Rhesus 6	Kidney	57.8	86.3	2.4	11.3	10530708
Rhesus 1	Heart	57.8	92.9	1.1	5.9	9116454
Rhesus 2	Heart	24.8	52.9	0.6	46.5	19394080
Rhesus 3	Heart	61.9	96.7	0.8	2.5	9093491
Rhesus 4	Heart	57.8	90.8	1.2	8	9824696
Rhesus 5	Heart	66.8	95	1.1	3.9	9018713
Orang 1	Brain	42.5	78.6	0.6	20.8	11307562
Orang 2	Brain	40.7	64.7	0.2	35	11449064
Orang 3	Liver	53.4	91.7	0.2	8.1	7111233
Orang 4	Liver	38.8	91.3	0.1	8.5	10302589
Gorilla 1	Brain	41.5	6.7	56.4	37	11931502
Gorilla 2	Brain	37.6	3.2	32.6	64.3	9534826
Gorilla 3	Liver	35	1.8	72.8	25.4	12400172
Gorilla 4	Liver	38.8	2.4	61.2	36.4	12018826

Column 1: individual information; column 2: tissue; column 3: fraction of reads that could be mapped perfectly to species corresponding genome; columns 4-6 are based on the reads that could be mapped to the corresponding species genome and contain how many of these reads could be aligned to known miRNAs (column 4), newly predicted miRNAs (column 5) and to neither of these 2 categories (column 6); column 7: total number of sequenced reads.

To identify miRNAs which are shared between all the primates studied here we examined miRNAs that are encoded in orthologous locations in all four primate species and in human. For the miRNAs present in miRBase (v.17) we found 60 miRNAs that are located in orthologous regions in human and the four non-human primate species. When we included the set of miRNAs predicted in this study we increased this number to 175 miRNAs. This set of miRNAs can be considered prediction of high confidence since they were known in human and either known or predicted by us in all other four primate species.

### Sequence identity

**Figure 2:**
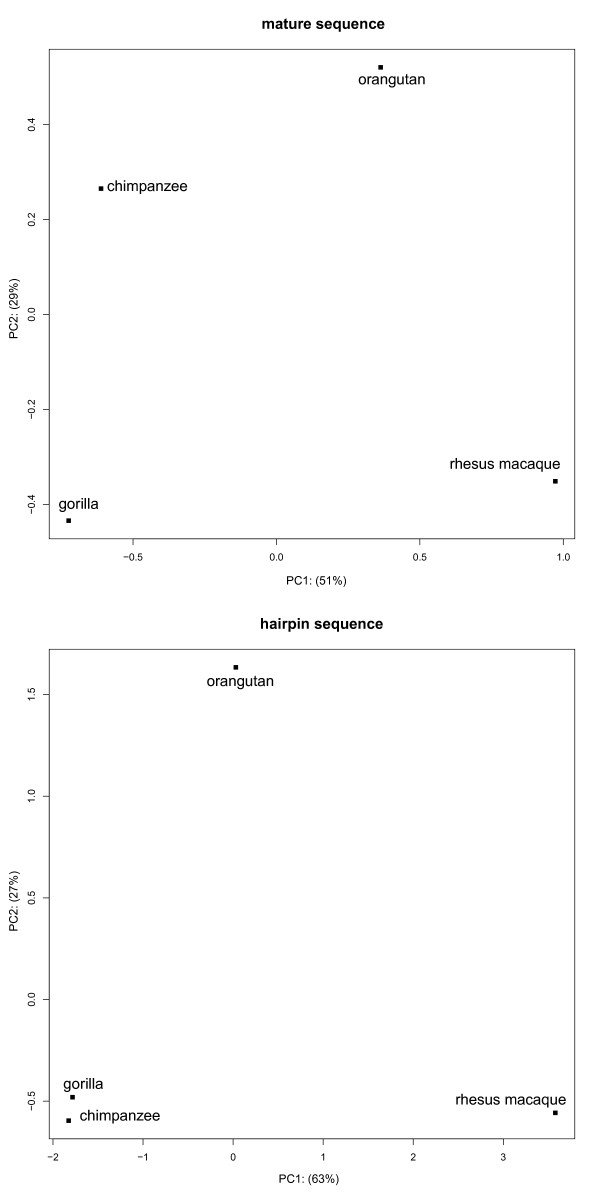
**Principle Component Analysis (PCA) using sequence similarity between mature (above) and hairpin (below) sequences**. The plots show the first two components of the corresponding PCAs and the amount of variance explained by each component.

All 60 of the known miRNAs present in all four species and human showed a high sequence identity i.e. the sequence is completely identical between the mature sequences for all of them. Using the set of 175 miRNAs we were able to reconstruct the expected phylogenetic relationships between the species studied for both the hairpin and the mature sequence. A principle component analysis on the sequence identity between hairpin sequences (Figure 2) shows a close relationship between chimpanzee and gorilla while both species are distant from orangutan and even more afar to rhesus macaque.

### Secondary structure

For some stages during their biogenesis miRNAs form a secondary structure that resembles a hairpin [30]. Since the endonuclease that processes miRNAs recognizes them based on their three-dimensional structure [30], the stability of the secondary structure can be considered a proxy for miRNA functionality and therefore for the reliability of miRNAs predictions. We used the minimum free energy (MFE) as a measure of structure stability. We found that the hairpins of predicted miRNAs are as stable as hairpins from known miRNAs, which is not unexpected given that the score calculated by miRDeep2 takes into account the stability of the miRNA hairpin secondary structure.

## Discussion

Although the genomes of multiple non-human primates have been sequenced, the functional annotation of the human genome remains the most complete among primates. This is the case for miRNAs annotated in miRBase, where the number of human miRNAs is double than miRNAs annotated in chimpanzee (the second-best annotated genome) [16,17]. In the present study we sequenced small RNA libraries from multiple individuals and tissues in four non-human primates in order to identify from expression data new miRNA genes. We identified these new miRNAs using miRDeep2 [27], which uses a model for miRNA precursor processing by Dicer to score miRNA predictions. Using this approach we predicted 47 new miRNAs in chimpanzee, 240 in gorilla, 55 in orangutan and 47 in rhesus macaque (Figure 1). We found that the secondary structures from our new miRNAs were as stable as miRNAs previously described in miRBase.

A similar number of new miRNAs were identified in chimpanzee, orangutan and rhesus macaque, whereas the number of new miRNA predictions in gorilla was much higher. While the genomes of the chimpanzee, orangutan and rhesus have been available for some time, and a number of miRNA studies in these species published, the gorilla genome has not yet been published and fully annotated [4,5,31], and no published description of miRNAs in gorilla - a requirement for inclusion of new miRNAs in miRBase - exists The majority of annotated miRNAs in the non-human primates are based on homology with human miRNAs [20-22]. However, the presence of a given locus in a genome is not a guarantee of its expression. We have, in this study, provided evidence of expression for 51% of the homology-based annotated miRNAs in gorilla, 49% in chimpanzee and 60% in rhesus macaque. We increased from 60 to 175 the number of miRNAs, which are located in orthologous regions in the four non-human primate genomes studied here and in human. This is a set of high confidence miRNAs based on homology, expression and miRNA biogenesis signatures.

In addition to the analysis of expression and folding, miRDeep incorporates a model of miRNA biogenesis, which makes its predictions more accurate than other software [27]. While the sequencing of small RNA libraries is now technically feasible, the accurate identification of novel miRNAs remains challenging. A pioneer study in primates sequenced small RNAs libraries from human and chimpanzee brains [24]. They predicted a large number (268 in human and 257 in chimpanzee) of new miRNAs in both species based on small RNA sequencing. Only few of these miRNAs have been included in miRBase, the public, curated repository for miRNAs (49 in human and 19 in chimpanzee). It is important to identify novel miRNAs accurately, and therefore particularly important to take into account the effect of genome quality and completeness on the ability to determine whether particular miRNAs are species-specific In primate comparisons the higher quality and completeness of the human genome means that miRNAs are frequently described as human-specific when in fact they are simply missed in related primate genomes due to sequence quality issues.

We sought to identify miRNAs that are expressed in tissue-specific manner. For species where we had samples from five tissues (chimpanzee and rhesus) we could say with more confidence that a given miRNA is tissue-specific than for the species where we had only two tissues (orangutan and gorilla). Brain was the tissue with both more miRNAs in total, and more tissue-specific miRNAs both in chimpanzee and marginally in rhesus. In orangutan and gorilla we could only identify miRNAs that are expressed mutually exclusively in either liver or brain. We found more miRNAs expressed exclusively in brain than in liver. This is in agreement with the fact that the miRNA repertoire in humans, chimpanzees and rhesus macaques is more diverse in brain compared to other tissues [29].

## Conclusion

We have sequenced small RNA libraries from multiple individuals and tissues from chimpanzee, gorilla, orangutan and rhesus macaque. We identified known miRNAs and used miRDeep2 to predict *de novo *microRNAs in these four primate species. Our new expression-based predictions increased the number of known miRNAs in all four species. In addition, we showed the first expression evidence for miRNAs that were previously only annotated by sequence homology with humans. Accurate annotation of miRNAs in multiple primate species provides a fundamental to carry out evolutionary, comparative and functional studies of miRNAs.

## Methods

### MiRNA samples

We sequenced 56 small RNA libraries (24 from chimpanzees, 24 from rhesus macaques, four from orangutan and four from gorilla). The chimpanzee and rhesus macaque samples have been published [29]. We added to this set eight samples from orangutan and gorilla (four liver and four brain samples from each species). All the individuals used in this study were adults and suffered sudden death that did not involve the tissues sampled. A description of the samples is available in Table 1.

### Library preparation and sequencing

We used the individuals presented in [29] including 24 chimpanzee and rhesus macaque samples. Additionally, we sequenced four gorilla and four orangutan samples from brain and liver (two from each species and tissue). Total RNA was prepared as described in the Illumina Inc. manual "Small RNA Sample Preparation Guide" (Part # 1004239 Rev. A Illumina Inc. San Diego). Illumina Genome Analyzer I and II sequencing runs were analyzed starting from raw intensities. A detailed summary about the platform each sample was sequenced on, how many cycles and which chemistry was used can be found in Table 2. Base calling and quality score calculation was performed for all runs using the IBIS base caller [32].

**Table 2:** Sequencing information.

Individual	Tissue	Sex	Platform	Chemistry	Cycles
Orang 1	Brain	Male	GA 1	V2	26
Orang 2	Brain	Female	GA 1	V2	36
Orang 3	Liver	Male	GA 2	V1	26
Orang 4	Liver	Male	GA 1	V1	36
Gorilla 1	Brain	Female	GA 1	V2	26
Gorilla 2	Brain	Female	GA 1	V2	36
Gorilla 3	Liver	Female	GA 2	V1	26
Gorilla 4	Liver	Female	GA 1	V1	36

### Sample composition and read annotation

Read alignments were performed using PatMaN [33] allowing no mismatches. We mapped reads against miRBase [16,17] version 17 and the corresponding species genomes - chimpanzee (panTro3), rhesus macaque (rheMac2), orangutan (ponAbe2) and the draft genome of gorilla (gorGor3).

### Sequence data

MiRNA data was uploaded to the European Nucleotide Archive hosted by the European Bioinformatics Institute with the study accession number ERP000973 and ArrayExpress with accession number E-MTAB-828.

### MiRNAs prediction

We used miRDeep2 prediction algorithm [27]. All reads from each species were used for the corresponding predictions. We excluded redundant predictions for the same genomic location and only kept the prediction with the highest score. We used the mapper module (mapper.pl) provided by miRDeep2 with the following parameters: -n -d -c -i -j -l 18 -m -k TCGTATGCCGTCTTCTGCTTG. We ran miRDeep2 with default parameters. Newly predicted miRNAs that were found in orthologous genomic regions in all four species were submitted to miRBase. Names were assigned by miRBase and are available in Table 3.

**Table 3:** Novel miRNAs

species	miRBase id	mature sequence	chromosome	miRDeep2 score
chimpanzee	ptr-mir-4423	AUAGGCACCAAAAAGCAACAA	1	24.7
chimpanzee	ptr-mir-3121	UAAAUAGAGUAGGCAAAGGACA	1	25919
chimpanzee	ptr-mir-3117	AUAGGACUCAUAUAGUGCCAGG	1	4.2
chimpanzee	ptr-mir-4742	UCAGGCAAAGGGAUAUUUACAGA	1	4.7
chimpanzee	ptr-mir-4428	CAAGGAGACGGGAACAUGGAGCC	1	5.2
chimpanzee	ptr-mir-4654	UGUGGGAUCUGGAGGCAUCUGGG	1	5.7
chimpanzee	ptr-mir-92b	UAUUGCACUCGUCCCGGCCUCC	1	9795.4
chimpanzee	ptr-mir-3127	AUCAGGGCUUGUGGAAUGGGAAG	2A	103.7
chimpanzee	ptr-mir-3132	UGGGUAGAGAAGGAGCUCAGA	2B	5.5
chimpanzee	ptr-mir-3129	GCAGUAGUGUAGAGAUUGGU	2B	92.4
chimpanzee	ptr-mir-378b	ACUGGACUUGGAGGCAGAAA	3	5.2
chimpanzee	ptr-mir-4446	CAGGGCUGGCAGUGAGAUGGG	3	5.3
chimpanzee	ptr-mir-3136	CUGACUGAAUAGGUAGGGUCA	3	5.5
chimpanzee	ptr-mir-3138	ACAGUGAGGUAGAGGGAGUG	4	148.4
chimpanzee	ptr-mir-3660	ACUGACAGGAGAGCGUUUUGA	5	120.4
chimpanzee	ptr-mir-378e	ACUGGACUUGGAGUCAGG	5	5
chimpanzee	ptr-mir-449c	AGGCAGUGUAUUGCUAGCGGCUGU	5	5.4
chimpanzee	ptr-mir-3943	UAGCCCCCAGGCUUCACUUGGCG	7	47.7
chimpanzee	ptr-mir-4660	UGCAGCUCUGGUGGAAAAUGGA	8	45124
chimpanzee	ptr-mir-3151	GGUGGGGCAAUGGGAUCAGGUG	8	500.7
chimpanzee	ptr-mir-3149	UUUGUAUGGAUAUGUGUGUGUA	8	5.3
chimpanzee	ptr-mir-4667	ACUGGGGAGCAGAAGGAGAACC	9	5.5
chimpanzee	ptr-mir-548e	AAAAACUGCGACUACUUUUG	10	5.4
chimpanzee	ptr-mir-3664	UCAGGAGUAAAGACAGAGU	11	5.6
chimpanzee	ptr-mir-1260b	AUCCCACCACUGCCACCAU	11	5.8
chimpanzee	ptr-mir-3165	AGGUGGAUGCAAUGUGACCUCA	11	5.9
chimpanzee	ptr-mir-1252	AGAAGGAAGUUGAAUUCAUU	12	4.6
chimpanzee	ptr-mir-200c	UAAUACUGCCGGGUAAUGAUGGA	12	5.8
chimpanzee	ptr-mir-655	AUAAUACAUGGUUAACCUCUU	14	246.1
chimpanzee	ptr-mir-3173	AAAGGAGGAAAUAGGCAGGCCA	14	344.5
chimpanzee	ptr-mir-2392	UAGGAUGGGGGUGAGAGGUG	14	5
chimpanzee	ptr-mir-4504	UGUGACAAUAGAGAUGAACAUGG	14	5.8
chimpanzee	ptr-mir-4510	UGAGGGAGUAGGAUGUAUGGU	15	4.2
chimpanzee	ptr-mir-4524a	UGAGACAGGCUUAUGCUGCUA	17	195.8
chimpanzee	ptr-mir-4743	UGGCCGGAUGGGACAGGAGGCA	18	5.4
chimpanzee	ptr-mir-320e	AAAAGCUGGGUUGAGAAGGUGA	19	4.5
chimpanzee	ptr-mir-548o	AAAAGUAAUUGCGGUUUUUGCC	20	105.8
chimpanzee	ptr-mir-3193	CUCCUGCGUAGGAUCUGAGGAG	20	4.7
chimpanzee	ptr-mir-3192	UCUGGGAGGUUGUAGCAGUGGA	20	5
chimpanzee	ptr-mir-3200	CACCUUGCGCUACUCAGGUCUG	22	270.9
chimpanzee	ptr-mir-23c	AUCACAUUGCCAGUGAUUACCC	X	4.4
chimpanzee	ptr-mir-2114	CGAGCCUCAAGCAAGGGACUUCA	X	50.6
chimpanzee	ptr-mir-767	UGCACCAUGGUUGUCUGAGCA	X	5.3
chimpanzee	ptr-mir-4536	UGUGGUAGAUAUAUGCACGA	X	5.3
chimpanzee	ptr-mir-222	AGCUACAUCUGGCUACUGGGUC	X	5.6
chimpanzee	ptr-mir-3937	ACAGGCGGCUGUAGCAAUGGGGGG	X	6.1
chimpanzee	ptr-mir-676	CUGUCCUAAGGUUGUUGAGU	X	79.5

gorilla	ggo-mir-135b	UAUGGCUUUUCAUUCCUAUGUGA	1	10.3
gorilla	ggo-mir-3605	GAUGAGGAUGGAUAGCAAGGAAG	1	1.1
gorilla	ggo-mir-29c	UAGCACCAUUUGAAAUCGGUUA	1	11813.8
gorilla	ggo-mir-197	UUCACCACCUUCUCCACCCAGC	1	119.9
gorilla	ggo-mir-92b	UAUUACACUCGUCCCGGCCUCC	1	1589.6
gorilla	ggo-mir-30e	UGUAAACAUCCUUGACUGGAAGC	1	3114.3
gorilla	ggo-mir-556	AUAUUACCAUUAGCUCAUCU	1	36.8
gorilla	ggo-mir-488	CCCAGAUAAUGGCACUCUCAA	1	4.7
gorilla	ggo-mir-320b	AGAAGCUGGGUUGAGAGGGCAA	1	5
gorilla	ggo-mir-190b	UGAUAUGUUUGAUAUUGGGUUG	1	5.1
gorilla	ggo-mir-429	UAAUACUGUCUGGUAAAACCG	1	5.3
gorilla	ggo-mir-760	CGGCUCUGGGUCUGUGGGGAG	1	5.4
gorilla	ggo-mir-1278	UAGUACUGUGCAUAUCAUCUA	1	5.6
gorilla	ggo-mir-551a	GCGACCCACUCUUGGUUUCCA	1	83
gorilla	ggo-mir-200b	UAAUACUGCCUGGUAAUGAUGAC	1	86.9
gorilla	ggo-mir-200a	UAACACUGUCUGGUAACGAUGU	1	99.7
gorilla	ggo-mir-4429	AAAAGCUGGGCUGAGAGGCGA	2A	1
gorilla	ggo-mir-3126	UGAGGGACAGAUGCCAGAAGCA	2A	5.3
gorilla	ggo-mir-1301	UUGCAGCUGCCUGGGAGUGACU	2A	5.5
gorilla	ggo-mir-3127	AUCAGGGCUUGUGGAAUGGGA	2A	5.6
gorilla	ggo-mir-26b	UUCAAGUAAUUCAGGAUAGGU	2B	15749.2
gorilla	ggo-mir-375	UUUGUUCGUUCGGCUCGCGUGA	2B	1.7
gorilla	ggo-mir-128	UCACAGUGAACCGGUCUCUU	2B	22571.1
gorilla	ggo-mir-149	UCUGGCUCCGUGUCUUCACUCCC	2B	357.8
gorilla	ggo-mir-3129	GCAGUAGUGUAGAGAUUGGU	2B	4
gorilla	ggo-mir-191	CAACGGAAUCCCAAAAGCAGC	3	13047.6
gorilla	ggo-let-7g	UGAGGUAGUAGUUUGUACAGU	3	134084.7
gorilla	ggo-mir-3923	AACUAGUAAUGUUGGAUUAGGGC	3	1.5
gorilla	ggo-mir-28	CACUAGAUUGUGAGCUCCUGGA	3	-4.8
gorilla	ggo-mir-4446	CAGGGCUGGCAGUGAGAUGGG	3	5.2
gorilla	ggo-mir-378b	ACUGGACUUGGAGGCAGAAAG	3	5.2
gorilla	ggo-mir-885	AGGCAGCGGGGUGUAGUGGA	3	5.7
gorilla	ggo-mir-551b	GCGACCCAUACUUGGUUUCAG	3	74.8
gorilla	ggo-mir-1255a	AGGAUGAGCAAAGAAAGUAGAU	4	122.2
gorilla	ggo-mir-548d	CAAAAACUGCAGUUACUUUUG	4	17.8
gorilla	ggo-mir-577	AUAGAUAAAAUAUUGGUACCUG	4	1.8
gorilla	ggo-mir-3138	ACAGUGAGGUAGAGGGAGUG	4	2.3
gorilla	ggo-mir-574	CACGCUCAUGCACACACCCACA	4	510.5
gorilla	ggo-mir-378e	ACUGGACUUGGAGUCAGGAC	5	0.5
gorilla	ggo-mir-3615	UCUCUCCGCUCCUCGCGGCUCGC	5	11.9
gorilla	ggo-mir-423	UGAGGGGCAGAGAGCGAGACUU	5	12767.2
gorilla	ggo-mir-4524a	UGAGACAGGCUUAUGCUGCUA	5	150
gorilla	ggo-mir-338	UCCAGCAUCAGUGAUUUUGUUGA	5	1509.7
gorilla	ggo-mir-193a	AACUGGCCUACAAAGUCCCAG	5	1740.8
gorilla	ggo-mir-1180	UUUCCGGCUCGCGUGGGUGUG	5	1.9
gorilla	ggo-mir-144	GGAUAUCAUCAUAUACUGUAAG	5	245.3
gorilla	ggo-mir-454	UAGUGCAAUAUUGCUUAUAGGGUU	5	4.9
gorilla	ggo-mir-152	UCAGUGCAUGACAGAACUUGG	5	5070.4
gorilla	ggo-mir-146a	UGAGAACUGAAUUCCAUGGGU	5	5.2
gorilla	ggo-mir-874	CUGCCCUGGCCCGAGGGACCGA	5	526.7
gorilla	ggo-mir-142	CCCAUAAAGUAGAAAGCACUA	5	5.3
gorilla	ggo-mir-1250	ACGGUGCUGGAUGUGGCCUU	5	5.4
gorilla	ggo-mir-4738	UGAAACUGGAGCGCCUGGAG	5	5.5
gorilla	ggo-mir-584	UUAUGGUUUGCCUGGGACUGA	5	5.8
gorilla	ggo-mir-1271	CUUGGCACCUAGCAAGCACUCA	5	58.5
gorilla	ggo-mir-378	ACUGGACUUGGAGUCAGAAGGCC	5	7592.3
gorilla	ggo-mir-340	UUAUAAAGCAAUGAGACUGAU	5	8919.2
gorilla	ggo-mir-877	GUAGAGGAGAUGGCGCAGGGGACA	6	1.5
gorilla	ggo-mir-30c	UGUAAACAUCCUACACUCUCAGC	6	1740.7
gorilla	ggo-mir-548b	CAAAAACCUCAGUUGCUUUUG	6	17.9
gorilla	ggo-mir-548a	AAAAGUAAUUGUGGUUUUUGC	6	30.4
gorilla	ggo-mir-133b	UUUGGUCCCCUUCAACCAGC	6	4
gorilla	ggo-mir-206	UGGAAUGUAAGGAAGUGUGUGG	6	5.4
gorilla	ggo-mir-1273c	GGCGACAAAACGAGACCCUG	6	8.4
gorilla	ggo-mir-671	UCCGGUUCUCAGGGCUCCACC	7	24.5
gorilla	ggo-mir-3943	UAGCCCCCAGGCUUCACUUGGCG	7	34
gorilla	ggo-mir-148a	UCAGUGCACUACAGAACUUUG	7	3957.5
gorilla	ggo-mir-339	UGAGCGCCUCGACGACAGAGCCG	7	429.6
gorilla	ggo-mir-592	UUGUGUCAAUAUGCGAUGAUG	7	45.6
gorilla	ggo-mir-548f	CAAAAGUGAUCGUGGUUUUUG	7	4.6
gorilla	ggo-mir-589	UGAGAACCACGUCUGCUCUGA	7	5.3
gorilla	ggo-mir-182	UUUGGCAAUGGUAGAACUCACA	7	5.4
gorilla	ggo-mir-590	GAGCUUAUUCAUAAAAGUGCAG	7	57.4
gorilla	ggo-mir-490	CAACCUGGAGGACUCCAUGCUG	7	73.8
gorilla	ggo-mir-335	UCAAGAGCAAUAACGAAAAAUG	7	785.9
gorilla	ggo-mir-486	UCCUGUACUGAGCUGCCCCGAG	8	1100
gorilla	ggo-mir-383	AGAUCAGAAGGUGAUUGUGGC	8	1642.2
gorilla	ggo-mir-3151	GGUGGGGCAAUGGGAUCAGGUG	8	18.3
gorilla	ggo-mir-598	UACGUCAUCGUUGUCAUCGUCA	8	5151.1
gorilla	ggo-mir-4660	UGCAGCUCUGGUGGAAAAUGGA	8	5.2
gorilla	ggo-mir-320a	AAAAGCUGGGUUGAGAGGGCGA	8	5.5
gorilla	ggo-mir-151a	UCGAGGAGCUCACAGUCUAG	8	5.6
gorilla	ggo-mir-455	GCAGUCCAUGGGCAUAUACAC	9	1166.5
gorilla	ggo-let-7f	UGAGGUAGUAGAUUGUAUAGU	9	1167727.6
gorilla	ggo-mir-873	GCAGGAACUUGUGAGUCUCC	9	197.5
gorilla	ggo-mir-27b	UUCACAGUGGCUAAGUUCUGC	9	2594.1
gorilla	ggo-mir-23b	AUCACAUUGCCAGGGAUUACCA	9	5
gorilla	ggo-mir-3927	CAGGUAGAUAUUUGAUAGGCA	9	6
gorilla	ggo-mir-491	AGUGGGGAACCCUUCCAUGAGGA	9	92.5
gorilla	ggo-mir-1287	UGCUGGAUCAGUGGUUCGAG	10	0.8
gorilla	ggo-mir-146b	UGAGAACUGAAUUCCAUAGGCUGU	10	10004.3
gorilla	ggo-mir-2110	UUGGGGAAGCGGCCGCUGAGUGA	10	1.4
gorilla	ggo-mir-346	UGUCUGCCCGCAUGCCUGCCUC	10	1.8
gorilla	ggo-mir-4484	GAAAAAGGCGGGAGAAGCCCCA	10	-2.5
gorilla	ggo-mir-202	AAGAGGUAUAGGGCAUGGGAAA	10	4.3
gorilla	ggo-mir-609	AGGGUGUUUCUCUCAUCUCUGG	10	4.3
gorilla	ggo-mir-548e	AAAAACUGCGACUACUUUUG	10	5.4
gorilla	ggo-mir-1296	UUAGGGCCCUGGCUCCAUCUCC	10	5.6
gorilla	ggo-mir-548c	AAAAGUACUUGCGGAUUUUG	11	12.7
gorilla	ggo-mir-34c	AGGCAGUGUAGUUAGCUGAUUG	11	1287.5
gorilla	ggo-mir-483	AAGACGGGAGGAAAGAAGGGAG	11	1967.6
gorilla	ggo-mir-4488	UAGGGGGCGGGCUCCGGCG	11	2
gorilla	ggo-mir-192	CUGACCUAUGAAUUGACAGCC	11	243338.1
gorilla	ggo-mir-34b	AGGCAGUGUCAUUAGCUGAUUG	11	28.3
gorilla	ggo-mir-210	CUGUGCGUGUGACAGCGGCUGA	11	323
gorilla	ggo-mir-675b	UGGUGCGGAGAGGGCCCACAGUG	11	41.1
gorilla	ggo-mir-139	UCUACAGUGCACGUGUCUCCAG	11	4363.3
gorilla	ggo-mir-1260b	AUCCCACCACUGCCACCA	11	5.6
gorilla	ggo-mir-326	CCUCUGGGCCCUUCCUCCAG	11	5.7
gorilla	ggo-mir-129	AAGCCCUUACCCCAAAAAGCA	11	7084.6
gorilla	ggo-mir-331	GCCCCUGGGCCUAUCCUAGAAC	12	1050.8
gorilla	ggo-mir-3612	AGGAGGCAUCUUGAGAAAUGG	12	12.5
gorilla	ggo-mir-1252	AGAAGGAAGUUGAAUUCAUU	12	16
gorilla	ggo-mir-148b	UCAGUGCAUCACAGAACUUUG	12	2086.5
gorilla	ggo-let-7i	UGAGGUAGUAGUUUGUGCUGU	12	25708.1
gorilla	ggo-mir-1228	GUGGGCGGGGGCAGGUGUGUGG	12	30.4
gorilla	ggo-mir-1291	GUGGCCCUGACUGAAGACCAGCA	12	5.3
gorilla	ggo-mir-1197	UAGGACACAUGGUCUACUUC	14	-0.3
gorilla	ggo-mir-370	GCCUGCUGGGGUGGAACCUGGUC	14	0.6
gorilla	ggo-mir-431	UGCAGGUCGUCUUGCAGGGCU	14	1
gorilla	ggo-mir-380	UAUGUAAUAUGGUCCACAUC	14	106
gorilla	ggo-mir-3545	UUGAACUGUUAAGAACCACUGG	14	12.6
gorilla	ggo-mir-433	AUCAUGAUGGGCUCCUCGGUG	14	1331
gorilla	ggo-mir-376a	AUCAUAGAGGAAAAUCCACG	14	156.3
gorilla	ggo-mir-655	AUAAUACAUGGUUAACCUCUU	14	158.8
gorilla	ggo-mir-379	UGGUAGACUAUGGAACGUAGG	14	1946
gorilla	ggo-mir-624	UAGUACCAGUACCUUGUGUUCA	14	2
gorilla	ggo-mir-409	AGGUUACCCGAGCAACUUUGCA	14	233
gorilla	ggo-mir-487a	AAUCAUACAGGGACAUCCAGU	14	245.1
gorilla	ggo-mir-495	AAACAAACAUGGUGCACUUCU	14	2528.9
gorilla	ggo-mir-543	AAACAUUCGCGGUGCACUUCU	14	260.4
gorilla	ggo-mir-432	UCUUGGAGUAGGUCAUUGGGUG	14	2631.8
gorilla	no id*^1^	AGGGGGAAAGUUCUAUAG	14	3.4
gorilla	ggo-mir-493	UUGUACAUGGUAGGCUUUCAU	14	38.4
gorilla	ggo-mir-889	UUAAUAUCGGACAACCAUUG	14	3.9
gorilla	ggo-mir-485	AGAGGCUGGCCGUGAUGAAU	14	3983.2
gorilla	ggo-mir-299	UGGUUUACCGUCCCACAUACA	14	446.3
gorilla	ggo-mir-494	UGAAACAUACACGGGAAACCUC	14	4.7
gorilla	ggo-mir-329b	AACACACCUGGUUAACCUCU	14	4.7
gorilla	ggo-mir-1185	AGAGGAUACCCUUUGUAUGU	14	5
gorilla	ggo-mir-496	UGAGUAUUACAUGGCCAAUC	14	5
gorilla	ggo-mir-487b	AAUCGUACAGGGUCAUCCACU	14	5.1
gorilla	ggo-mir-127	UCGGAUCCGUCUGAGCUUGGC	14	5.2
gorilla	ggo-mir-323b	CCCAAUACACGGUCGACCUC	14	5.3
gorilla	ggo-mir-337	GAACGGCUUCAUACAGGAG	14	5.3
gorilla	ggo-mir-668	AUGUCACUCGGCUCGGCCCAC	14	5.3
gorilla	ggo-mir-342	UCUCACACAGAAAUCGCACCCG	14	5.4
gorilla	ggo-mir-1193	GGGAUGGUAGACCGGUGACGUGC	14	5.4
gorilla	ggo-mir-376c	AACAUAGAGGAAAUUCCACG	14	558
gorilla	ggo-mir-3173	AAAGGAGGAAAUAGGCAGGCCAG	14	5.7
gorilla	ggo-mir-654	UGGUGGGCCGCAGAACAUGUGC	14	58.5
gorilla	ggo-mir-411	AUAGUAGACCGUAUAGCGUACG	14	587.6
gorilla	ggo-mir-656	AAUAUUAUACAGUCAACCUC	14	59.4
gorilla	ggo-mir-410	AAUAUAACACAGAUGGCCUG	14	644.2
gorilla	ggo-mir-376b	AUCAUAGAGGAAAAUCCAUG	14	71.1
gorilla	ggo-mir-377	AUCACACAAAGGCAACUUUUG	14	83.6
gorilla	ggo-mir-381	UAUACAAGGGCAAGCUCUCUG	14	86.1
gorilla	ggo-mir-345	GCUGACUCCUAGUCCAGGGCUCG	14	88.9
gorilla	ggo-mir-323a	CACAUUACACGGUCGACCUC	14	894
gorilla	ggo-mir-628	AUGCUGACAUAUUUACUAGAGG	15	141.7
gorilla	ggo-mir-1179	AAGCAUUCUUUCAUUGGUUGG	15	27.1
gorilla	ggo-mir-4510	UGAGGGAGUAGGAUGUAUGGU	15	4.7
gorilla	ggo-mir-1266	CCUCAGGGCUGUAGAACAGGGCUG	15	5.9
gorilla	ggo-mir-629	UGGGUUUAUGUUGGGAGAACU	15	78.2
gorilla	ggo-mir-1343	CUCCUGGGGCCCGCACUC	16	1
gorilla	ggo-mir-484	UCAGGCUCAGUCCCCUCCCGA	16	1.1
gorilla	ggo-mir-328	CUGGCCCUCUCUGCCCUUCCG	16	116.1
gorilla	ggo-mir-193b	CGGGGUUUUGAGGGCGAGAUGA	16	1197.1
gorilla	ggo-mir-940	AAGGCAGGGCCCCCGCUCCCC	16	1.9
gorilla	ggo-mir-138	AGCUGGUGUUGUGAAUCAGGCCG	16	3411
gorilla	ggo-mir-365a	UAAUGCCCCUAAAAAUCCUUA	16	698
gorilla	ggo-mir-140	ACCACAGGGUAGAACCACGGAC	16	97632.3
gorilla	ggo-mir-324	CGCAUCCCCUAGGGCAUUGGUG	17	550.3
gorilla	ggo-mir-497	CAGCAGCACACUGUGGUUUG	17	5.6
gorilla	ggo-mir-4520b	UUUGGACAGAAAACACGCAGG	17	5.6
gorilla	ggo-mir-887	GUGAACGGGCGCCAUCCCGAGGCU	17	81.3
gorilla	ggo-mir-22	AAGCUGCCAGUUGAAGAACUG	17	8262.6
gorilla	ggo-mir-582	UUACAGUUGUUCAACCAGUUAC	17	86.1
gorilla	ggo-mir-4529	UCAUUGGACUGCUGAUGGCCUG	18	0.8
gorilla	ggo-mir-122	UGGAGUGUGACAAUGGUGUUUG	18	2545110.2
gorilla	ggo-mir-4743	UGGCCGGAUGGGACAGGAGGCA	18	5.4
gorilla	ggo-mir-1	UGGAAUGUAAAGAAGUAUGUA	18	54001.2
gorilla	ggo-mir-517c	AUCGUGCAUCCCUUUAGAGUG	19	3
gorilla	ggo-mir-516b	AUCUGGAGGUAAGAAGCACUU	19	3.9
gorilla	ggo-mir-371b	ACUCAAAAGAUGGCGGCACUU	19	5.3
gorilla	ggo-mir-330	GCAAAGCACACGGCCUGCAGAGA	19	5.4
gorilla	ggo-mir-769	UGAGACCUCUGGGUUCUGAGC	19	545.2
gorilla	ggo-mir-125a	UCCCUGAGACCCUUUAACCUG	19	5.5
gorilla	ggo-mir-641	AAAGACAUAGGAUAGAGUCACC	19	6
gorilla	ggo-mir-181d	AACAUUCAUUGUUGUCGGUGGGU	19	6323.7
gorilla	ggo-mir-150	UCUCCCAACCCUUGUACCAGUG	19	64.7
gorilla	ggo-let-7e	UGAGGUAGGAGGUUGUAUAGU	19	86198.3
gorilla	ggo-mir-1289	UGGAAUCCAGGAAUCUGCAUUU	20	5.2
gorilla	ggo-mir-499a	UUAAGACUUGCAGUGAUGUU	20	5.5
gorilla	ggo-mir-296	AGGGUUGGGUGGAGGCUCUCC	20	6.2
gorilla	ggo-let-7c	UGAGGUAGUAGGUUGUAUGGU	21	270515.7
gorilla	ggo-mir-155	UUAAUGCUAAUCGUGAUAGGGG	21	5.3
gorilla	ggo-mir-1306	ACGUUGGCUCUGGUGGUGAUG	22	1.1
gorilla	ggo-mir-1286	UGCAGGACCAAGAUGAGCCCU	22	1.3
gorilla	ggo-let-7b	UGAGGUAGUAGGUUGUGUGGU	22	224101.1
gorilla	ggo-mir-1249	ACGCCCUUCCCCCCCUUCUUCA	22	29.3
gorilla	ggo-let-7a	UGAGGUAGUAGGUUGUAUAGU	22	523694.4
gorilla	ggo-mir-130b	CAGUGCAAUGAUGAAAGGGCA	22	548.3
gorilla	ggo-mir-185	UGGAGAGAAAGGCAGUUCCUGA	22	9137.4
gorilla	ggo-mir-18b	UAAGGUGCAUCUAGUGCAGU	X	-0.1
gorilla	ggo-mir-4536	UAUCGUGCAUAUAUCUACCACA	X	0.4
gorilla	ggo-mir-508	ACUGUAGCCUUUCUGAGUAGA	X	0.7
gorilla	ggo-mir-374b	AUAUAAUACAACCUGCUAAGUG	X	1006.8
gorilla	ggo-mir-532	CAUGCCUUGAGUGUAGGACCG	X	1105.2
gorilla	ggo-mir-542	UGUGACAGAUUGAUAACUGAAA	X	121
gorilla	ggo-mir-450b	UUUUGCAAUAUGUUCCUGAAUA	X	16
gorilla	ggo-mir-502a	AAUGCACCUGGGCAAGGAUUCA	X	164
gorilla	ggo-mir-503	UAGCAGCGGGAACAGUUCUGCAG	X	180.3
gorilla	ggo-mir-504	GACCCUGGUCUGCACUCUA	X	2
gorilla	ggo-mir-188	CAUCCCUUGCAUGGUGGAGGGUG	X	20.1
gorilla	ggo-mir-424	CAGCAGCAAUUCAUGUUUUGA	X	2017.9
gorilla	ggo-mir-509	UACUGCAGACGUGGCAAUCAUG	X	20.9
gorilla	ggo-mir-660	UACCCAUUGCAUAUCGGAGUUG	X	247.5
gorilla	ggo-mir-652	AAUGGCGCCACUAGGGUUGUG	X	291.5
gorilla	ggo-mir-363	AAUUGCACGGUAUCCAUCUGUAA	X	362.8
gorilla	ggo-mir-676	CUGUCCUAAGGUUGUUGAGUUG	X	4
gorilla	ggo-mir-374a	CUUAUCAGAUUGUAUUGUAAU	X	414.8
gorilla	ggo-mir-105	CCACGGAUGUUUGAGCAUGUG	X	-4.4
gorilla	ggo-mir-23c	AUCACAUUGCCAGUGAUUACCC	X	4.4
gorilla	ggo-mir-421	AUCAACAGACAUUAAUUGGGCG	X	5
gorilla	ggo-mir-20b	CAAAGUGCUCAUAGUGCAGGUAG	X	5
gorilla	ggo-mir-651	UUUAGGAUAAGCUUGACUUUUG	X	5
gorilla	ggo-mir-452	AACUGUUUGCAGAGGAAACUGA	X	5.2
gorilla	ggo-mir-767	UGCACCAUGGUUGUCUGAGCA	X	5.3
gorilla	ggo-mir-502b	AUGCACCUGGGCAAGGAUUCUGA	X	5.3
gorilla	ggo-mir-505	GUCAACACUUGCUGGUUUCC	X	5.4
gorilla	ggo-mir-1298	UUCAUUCGGCUGUCCAGAUG	X	5.4
gorilla	ggo-mir-222	AGCUACAUCUGGCUACUGGGUC	X	5.6
gorilla	ggo-mir-361	UUAUCAGAAUCUCCAGGGGUAC	X	615.7
gorilla	ggo-mir-450a	UUUUGCGAUGUGUUCCUAAUA	X	69.1
gorilla	ggo-mir-448	UUGCAUAUGUAGGAUGUCCCA	X	70
gorilla	ggo-mir-362	AACACACCUAUUCAAGGAUUCA	X	70.8
gorilla	ggo-mir-766	ACUCCAGCCCCACAGCCUCAGC	X	72.8
gorilla	ggo-mir-1264	ACAAGUCUUAUUUGAGCACCUG	X	7.8
gorilla	ggo-mir-1277	UACGUAGAUAUAUAUGUAUUU	X	93.5

orangutan	ppy-mir-4427	UCUGAAUAGAGUCUGAAGAG	1	0.2
orangutan	ppy-mir-3121	UAAAUAGAGUAGGCAAAGGACA	1	1.2
orangutan	ppy-mir-1976	CUCCUGCCCUCCUUGCUGUAG	1	3.8
orangutan	ppy-mir-4774	UCUGGUAUGUAGUAGGUAAUAA	2B	2.1
orangutan	ppy-mir-4782	UUCUGGAUAUGAAGACAAUCA	2B	3.2
orangutan	ppy-mir-4791	UGGAUAUGAUGACUGAAA	3	0.8
orangutan	ppy-mir-4446	CAGGGCUGGCAGUGAGAUGGG	3	2829
orangutan	ppy-mir-4796	UAAAGUGGCAGAGUAUAGACACA	3	3.3
orangutan	ppy-mir-378b	ACUGGACUUGGAGGCAGAAAG	3	5.3
orangutan	ppy-mir-4788	ACGGACCAGCUAAGGGAGGCAU	3	5.9
orangutan	ppy-mir-3938	AAUUCCCUUGUAGAUAACCUGG	3	8.5
orangutan	ppy-mir-4798	UUCGGUAUACUUUGUGAAUUGG	4	11.1
orangutan	ppy-mir-4451	UGGUAGAGCUGAGGACAG	4	4.6
orangutan	ppy-mir-3661	UGACCUGGGACUCGGAUAGCUGC	5	1.5
orangutan	ppy-mir-548h	AAAAGUAAUUGCGGUUUUUG	5	23.7
orangutan	ppy-mir-4637	UACUAACUGCAGAUUCAAGUGA	5	3
orangutan	ppy-mir-378e	ACUGGACUUGGAGUCAGG	5	4.1
orangutan	ppy-mir-3912	UAACGCAUAAUAUGGACAUG	5	4.5
orangutan	ppy-mir-548f	CAAAAACUGUAAUUACUUUUG	5	5.1
orangutan	ppy-mir-3660	CACUGACAGGAGAGCAUUUUGA	5	5.3
orangutan	ppy-mir-548a	AAAAGUAAUUGUGGUUUUUG	6	4.9
orangutan	ppy-mir-1273e	GAGGCAGGAGAAUCGCUUG	6	5
orangutan	ppy-mir-3934	UCAGGUGUGGAAUCUGAGGCA	6	5.3
orangutan	ppy-mir-3145	AACUCCAAGCAUUCAAAACUCA	6	5.4
orangutan	ppy-mir-3943	UAGCCCCCAGGCUUCACUUGGCG	7	22.2
orangutan	ppy-mir-4667	UGACUGGGGAGCAGAAGGAGA	9	1.6
orangutan	ppy-mir-3154	CAGAAGGGGAGUUGGGAGCAG	9	1.9
orangutan	ppy-mir-4672	ACACAGCUGGACAGAGGGACGA	9	4.8
orangutan	ppy-mir-2861	GGCGGCGGGCGUCGGGCG	9	6
orangutan	ppy-mir-2278	GAGGGCAGUGUGUGUUGUGUGG	9	8.8
orangutan	ppy-mir-4484	AAAAAGGCGGGAGAAGCCCCG	10	3.9
orangutan	ppy-mir-548e	AAAACGGUGACUACUUUUGCA	10	4.8
orangutan	ppy-mir-202	UUCCUAUGCAUAUACUUCUU	10	49.7
orangutan	ppy-mir-3155a	CAGGCUCUGCAGUGGGAACGGA	10	6.1
orangutan	ppy-mir-548c	AAAAGUACUUGCGGAUUUUG	11	5
orangutan	ppy-mir-1260b	AUCCCACCACUGCCACCA	11	5.5
orangutan	ppy-mir-3170	CUGGGGUUCUGAGACAGACAG	13	2.4
orangutan	ppy-mir-151b	UCCAGGAGCUCACAGUCUAG	14	2.6
orangutan	ppy-mir-1193	GGGAUGGUAGACCGGUGACGUGC	14	5
orangutan	ppy-mir-3173	AAGGAGGAAAUAGGCAGGCCAG	14	5.8
orangutan	ppy-mir-3174	UAGUGAGUUAGAGAUGCAGAGC	15	1.7
orangutan	ppy-mir-4515	AGGACUGGACUCCCGGCGGC	15	2.9
orangutan	ppy-mir-10a	UACCCUGUAGAUCCGAAUUUG	17	4.3
orangutan	ppy-mir-454	UAGUGCAAUAUUGCUUAUAGGG	17	5
orangutan	ppy-mir-4520a	UGGACAGAAAACACGCAGGAAG	17	5.2
orangutan	ppy-mir-152	UCAGUGCAUGACAGAACUUGG	17	8232.8
orangutan	ppy-mir-4526	GCUGACAGCAGGGCCGGCCAC	18	2.8
orangutan	ppy-mir-4529	AUUGGACUGCUGAUGGCCUG	18	3.6
orangutan	ppy-mir-4743	UGGCCGGAUGGGACAGGAGGCA	18	5.4
orangutan	ppy-mir-3188	AGAGGCUUUGUGCGGACUCGG	19	1.1
orangutan	ppy-mir-3940	CAGCCCGGAUCCCAGCCCACUCA	19	1.5
orangutan	ppy-mir-320e	AAAAGCUGGGUUGAGAAGGUGA	19	4.6
orangutan	ppy-mir-3617	AAAGACAUAGUUGCAAGAUGGG	20	1.6
orangutan	ppy-mir-378d	ACUGGACUUGGAGUCAGA	X	4.3
orangutan	ppy-mir-676	CCGUCCUAAGGUUGUUGAGUUG	X	5.1

rhesus macaque	mml-mir-1255b	UACGGAUAAGCAAAGAAAGUGG	1	2.1
rhesus macaque	mml-mir-320b	AAAAGCUGGGUUGAGAGGGCAA	1	5.1
rhesus macaque	mml-mir-3122	GUUGGGACAAGAGAACGGUCU	1	5.5
rhesus macaque	mml-mir-1262	UGAUGGGUGAAUUUGUAGAAGG	1	647.1
rhesus macaque	mml-mir-4446	CAGGGCUGGCAGUGAGAUGGG	2	26007.7
rhesus macaque	mml-mir-1284	UCUGUACAGACCCUGGCUUU	2	4.5
rhesus macaque	mml-mir-4796	AAGUGGCAGAGUGUAGACACAA	2	5.9
rhesus macaque	mml-mir-3146	CAUGCUAGAACAGAAAGAAUGGG	3	5
rhesus macaque	mml-mir-4650	UGGAAGGUAGAAUGAGGCCUGAU	3	5.8
rhesus macaque	mml-mir-3145	UAUUUUGAGUGUUUGGAAUUGA	4	4.8
rhesus macaque	mml-mir-1243	AAACUGGAUCAAUUAUAGGAG	5	17.7
rhesus macaque	mml-mir-378d	ACUGGACUUGGAGUCAGAAGCA	5	4.8
rhesus macaque	mml-mir-3140	AAGAGCUUUUGGGAAUUCAGG	5	5.3
rhesus macaque	mml-mir-1255a	AGGAUGAGCAAAGGAAGUAGU	5	5.7
rhesus macaque	mml-mir-4803	UAACAUAAUAGUGUGGACUGA	6	5.6
rhesus macaque	mml-mir-1271	CUUGGCACCUAGCAAGCACUCA	6	980.3
rhesus macaque	mml-mir-1179	AAGCAUUCUUUCAUUGGUUGG	7	16.9
rhesus macaque	mml-mir-1185	AGAGGAUACCCUUUGUAUGU	7	5.2
rhesus macaque	mml-mir-3173	GAAGGAGGAAACAGGCAGGCCAG	7	5.8
rhesus macaque	mml-mir-4716	AAGGGGGAAGGACACAUGGAGA	7	6.1
rhesus macaque	mml-mir-3151	ACGGGUGGCGCAAUGGGAUCAG	8	223.8
rhesus macaque	mml-mir-1296	UUAGGGCCCUGGCUCCAUCUCCU	9	5.5
rhesus macaque	mml-mir-1249	ACGCCCUUCCCCCCCUUCUUCA	10	118
rhesus macaque	mml-mir-3200	CACCUUGCGCUACUCAGGUCUG	10	202.6
rhesus macaque	mml-mir-1258	AGUUAGGAUUAGGUCGUGGAA	12	5.9
rhesus macaque	mml-mir-217b	UACUGCAUCAGGAACUGAUUGGA	13	4.3
rhesus macaque	mml-mir-1260b	AUCCCACCACUGCCACCA	14	5.6
rhesus macaque	mml-mir-1304	UUCGAGGCUACAAUGAGAUGUG	14	5.8
rhesus macaque	no id*^2^	CCAGGCUGGAGUGCAGUGG	15	4.1
rhesus macaque	mml-mir-873	GCAGGAACUUGUGAGUCUCC	15	4275.6
rhesus macaque	mml-mir-4667	ACUGGGGAGCAGAAGGAGAAC	15	5.5
rhesus macaque	mml-mir-3927	CAGGUAGAUAUUUGAUAGGCA	15	6.1
rhesus macaque	mml-mir-1250	ACGGUGCUGAAUGUGGCCUU	16	5.6
rhesus macaque	mml-mir-320c	AAAAGCUGGGUUGACAGGGUAA	18	3.8
rhesus macaque	mml-mir-4743	UGGCCGGAUGGGACAGGAGGCA	18	5.3
rhesus macaque	mml-mir-518d	CUCUAGAGGAAAGCGCUUACUG	19	103
rhesus macaque	mml-mir-517c	AUCGUGCAGCCUUUUAGAGUG	19	106.7
rhesus macaque	mml-mir-519e	UUCUCCAAUGGGAAGCACCUUC	19	132.7
rhesus macaque	mml-mir-1283	CUACAAAGGAAAGCACUUUC	19	4.9
rhesus macaque	mml-mir-1323	UCAAAACUGAGGGGCAUUUUC	19	6232.9
rhesus macaque	mml-mir-1298	UUCAUUCGGCUGUCCAGAUGUA	X	198.4
rhesus macaque	mml-mir-891b	UGCAACGAACUUGAGCCAUUGA	X	24.7
rhesus macaque	mml-mir-2114	CGAGCCUCAAGCAAGGGACUUC	X	25.3
rhesus macaque	mml-mir-4536	UGUGGUAGAUAUAUGCACGA	X	4.2
rhesus macaque	mml-mir-1277	UACGUAGAUAUAUAUGUAUUU	X	543.7
rhesus macaque	mml-mir-676	CCGUCCUAAGGUUGUUGAGU	X	766.4
rhesus macaque	mml-mir-514b	AUUGACACCUCUGUGAGUAGA	X	997.4

*^1,2 ^miRBase did not provide names due to ambiguous N bases in the hairpin sequence or missing relationships to existing miRNAs in the database.

### Orthology of miRNAs

We identified orthologous regions starting from human hg19-based miRBase (version 17) hairpin locations [16,17]. The genome coordinates were transferred to hg18 coordinates using liftOver [34] with the 95% identity cutoff. Human mature sequences from miRBase were aligned to the human genome (hg18) and their corresponding hairpin sequences were assigned by overlapping genome coordinates using intersectBed from Bedtools [35]. All other primate miRNA mature sequences (known and predicted) were aligned against the corresponding genome and their genome locations were transferred to hg18 coordinates. The mature miRNA sequences found in the other primates that overlapped with human coordinates were defined as orthologous. The corresponding primate hairpin sequence was obtained by transferring the human genome hairpin coordinates to the corresponding primate genome. We excluded regions where liftOver was unable to identify an orthologous region.

### Tissue specificity

MiRNAs were defined to be tissue specific when less than 5% of reads map to other tissues. This means that at least 80% of the perfectly aligned reads in chimpanzee and rhesus macaque (where we have reads from 4 tissues), and 95% of the perfectly aligned reads in gorilla and orangutan (where we have reads from 2 tissues) that were used for the prediction of the miRNA came from one tissue.

### Sequence comparison

Sequence identity of miRNAs (mature/hairpin) in orthologous regions was computed using the multiple sequence alignment tool MUSCLE [36] and the *identity *function of the R package bio3d [37].

### Secondary structure analysis

We calculated the minimum free energy (MFE) of known and predicted hairpin sequences by using RNAfold algorithm with default parameters [38]. The MFE for each group of annotated/predicted miRNAs was computed by averaging the MFEs.

## Authors' contributions

MD: Conceived and designed the experiments, analyzed the data, contributed reagents/materials/analysis tools, wrote the paper. BN: Performed the experiments. EL: Performed the experiments. HAB: Conceived and designed the experiments, analyzed the data, contributed reagents/materials/analysis tools, wrote the paper. JK: Conceived and designed the experiments, contributed reagents/materials/analysis tools, wrote the paper. All authors read and approved the final manuscript.
